# Impact of long-course neoadjuvant radiation on postoperative low anterior resection syndrome and stoma status in rectal cancer: long-term functional follow-up of a randomized clinical trial

**DOI:** 10.1093/bjsopen/zrac127

**Published:** 2022-11-02

**Authors:** Siqi He, Jinquan Zhang, Runxian Wang, Li Li, Lishuo Shi, Donglin Ren, Jianping Wang, Yanhong Deng, Ruoxu Dou

**Affiliations:** Department of Gastrointestinal Surgery, The Fifth Affiliated Hospital of Sun Yat-sen University, Zhuhai, Guangdong, P. R. China; Department of Colorectal Surgery, The Sixth Affiliated Hospital, Sun Yat-sen University, Guangzhou, Guangdong, P. R. China; Guangdong Provincial Key Laboratory of Colorectal and Pelvic Floor Diseases, The Sixth Affiliated Hospital, Sun Yat-sen University, Guangzhou, Guangdong, P. R. China; Department of Colorectal Surgery, The Sixth Affiliated Hospital, Sun Yat-sen University, Guangzhou, Guangdong, P. R. China; Guangdong Provincial Key Laboratory of Colorectal and Pelvic Floor Diseases, The Sixth Affiliated Hospital, Sun Yat-sen University, Guangzhou, Guangdong, P. R. China; Department of Gastrointestinal Surgery, The Fifth Affiliated Hospital of Sun Yat-sen University, Zhuhai, Guangdong, P. R. China; Pelvic Floor Center, The Sixth Affiliated Hospital, Sun Yat-sen University, Guangzhou, Guangdong, P. R. China; Clinical Research Center, The Sixth Affiliated Hospital, Sun Yat-sen University, Guangzhou, Guangdong, P. R. China; Pelvic Floor Center, The Sixth Affiliated Hospital, Sun Yat-sen University, Guangzhou, Guangdong, P. R. China; Department of Anorectal Surgery, The Sixth Affiliated Hospital, Sun Yat-sen University, Guangzhou, Guangdong, P. R. China; Department of Colorectal Surgery, The Sixth Affiliated Hospital, Sun Yat-sen University, Guangzhou, Guangdong, P. R. China; Guangdong Provincial Key Laboratory of Colorectal and Pelvic Floor Diseases, The Sixth Affiliated Hospital, Sun Yat-sen University, Guangzhou, Guangdong, P. R. China; Guangdong Provincial Key Laboratory of Colorectal and Pelvic Floor Diseases, The Sixth Affiliated Hospital, Sun Yat-sen University, Guangzhou, Guangdong, P. R. China; Department of Medical Oncology, The Sixth Affiliated Hospital, Sun Yat-sen University, Guangzhou, Guangdong, P. R. China; Department of Gastrointestinal Surgery, The Fifth Affiliated Hospital of Sun Yat-sen University, Zhuhai, Guangdong, P. R. China; Department of Colorectal Surgery, The Sixth Affiliated Hospital, Sun Yat-sen University, Guangzhou, Guangdong, P. R. China; Guangdong Provincial Key Laboratory of Colorectal and Pelvic Floor Diseases, The Sixth Affiliated Hospital, Sun Yat-sen University, Guangzhou, Guangdong, P. R. China

## Abstract

**Background:**

Neoadjuvant radiation has been increasingly associated with postoperative bowel dysfunction, including low anterior resection syndrome (LARS). Although permanent stoma often results from severe bowel dysfunction and significantly impacts quality of life, the presence of stoma paradoxically excludes patients from functional follow-up. Hence, stoma status is rarely reported along with LARS, while assessment of both is essential for the comprehensive evaluation of bowel dysfunction in long-term survivors of rectal cancer.

**Method:**

Patients enrolled into the Neoadjuvant FOLFOX6 Chemotherapy with or without Radiation in Rectal Cancer (FOWARC) multicentre randomized clinical trial were randomized to receive long-course neoadjuvant chemoradiotherapy (nCRT) or chemotherapy (nCT) followed by sphincter-saving proctectomy and longitudinal follow-up. The primary outcome of the trial was disease-free survival. LARS score and stoma status were assessed secondarily for postoperative bowel function in the largest single-centre cohort of the trial.

**Results:**

Overall, 327 patients with locally advanced rectal cancer were enrolled in the original trial and 203 responded after a median follow-up of 83.4 months, of whom 24 (11.8 per cent) had persistent stoma, and 48 patients (23.6 per cent) reported major LARS. Compared with the nCT group, the nCRT group reported more persistent stomas (16.5 per cent *versus* 4.9 per cent, *P* = 0.014), and more major LARS in patients without persistent stoma (34.7 per cent *versus* 16.7 per cent, *P* = 0.003). The combined prevalence of persistent stoma and major LARS was significantly higher in the nCRT group (45.5 per cent *versus* 20.7 per cent, *P* < 0.001). Long-course neoadjuvant radiation (OR 2.20, 95 per cent c.i. 1.10 to 4.40, *P* = 0.027), height of anastomosis (OR 0.74, 95 per cent c.i. 0.61 to 0.91, *P* = 0.004), and anastomotic leak (OR 4.97, 95 per cent c.i. 2.24 to 11.05, *P* < 0.001) were associated with persistent stoma and major LARS in multivariate analysis.

**Conclusion:**

More than one-third of patients receiving sphincter-saving proctectomy reported major LARS or persistent stoma at long-term follow-up. Long-course neoadjuvant radiation, low anastomosis, and postoperative leak are independent risk factors for persistent stoma and major LARS.

## Teaser

This study aimed to comprehensively assess the bowel function status and the impact of radiation in long-term survivors of rectal cancer in a randomized clinical trial. More than one-third of patients receiving sphincter-saving proctectomy reported major LARS or persistent stoma at long-term follow-up. Long-course neoadjuvant radiation is an independent risk factor for persistent stoma and major LARS.

## Introduction

Total mesorectal excision (TME) and multidisciplinary treatment have markedly improved survival of patients with rectal carcinoma^1,2^. Better understanding of resection margins and advancement on anastomotic techniques have allowed more sphincter-preserving proctectomy without oncological compromise^3^; however, up to 80 per cent of the patients with restored bowel continuity experience bowel dysfunction such as incontinence, frequent bowel movements, clustering of stools, and urgency, collectively known as low anterior resection syndrome (LARS), with an impact on quality of life^4,5^. In addition, persistent stoma remains a problem, not only after abdominoperineal resection (APR), but also from unreversed diverting stoma or new stoma caused by anastomotic complications, severe bowel dysfunction, or local recurrence^6–8^. Together, LARS and persistent stoma continue to impact the quality of life (QoL) of rectal cancer survivors.

Although neoadjuvant radiation is a standard treatment for locally advanced rectal cancer, it has failed to translate the benefit of local control into overall survival^1^. Meanwhile, radiotherapy has been associated with impaired postoperative bowel function^9,10^. Neoadjuvant radiation was associated with worse LARS score and QoL in a post hoc analysis of the FOWARC randomized clinical trial conducted by our group^11,12^. Of note, questionnaires such as LARS score^13^ do not apply to patients with persistent stoma, the inclusion of whom is necessary for a comprehensive analysis of functional outcome. To address this issue, the impact of radiation on major LARS and persistent stoma on the same FOWARC cohort after long-term follow-up were analysed.

## Methods

### Patients and characteristics

Patients were recruited from the largest single-centre cohort of the Neoadjuvant FOLFOX6 Chemotherapy with or without Radiation in Rectal Cancer (FOWARC) multicentre randomized clinical trial (registration number NCT01211210; http://www.clinicaltrials.gov) as previously described^14^. Briefly, patients diagnosed with rectal adenocarcinoma staged II or III were randomized to receive neoadjuvant fluorouracil plus radiotherapy; mFOLFOX6 (modified fluorouracil, leucovorin, and oxaliplatin) chemotherapy plus radiotherapy; or mFOLFOX6 alone, before undergoing TME resection and adjuvant chemotherapy from 2010 to 2015. A radiation dose of 46.0–50.4 Gy was delivered in 23–28 fractions to the primary tumour and to mesorectal, presacral, and internal iliac lymph nodes. APR was performed for patients with involved levator ani or external anal sphincter, or for selective patients with ultra-low tumour (less than 3 cm from anal verge) depending on surgeon’s experience and patient’s preference. For patients undergoing restorative proctectomy, a diverting stoma was constructed at surgeon’s discretion. Anastomotic leak was defined as communication between the intra- and extraluminal compartments^15^, and was confirmed by pus or faecal discharge from the pelvic drain, CT, MRI, colonoscopy, or re-laparotomy. In addition, defecography before reversal of ileostomy (2–3 months after proctectomy, if applicable) were routinely performed, as well as CT at the 6-month postoperative follow-up, to detect any subclinical leak. Any abnormalities found during the examination, especially anastomotic leakage, would delay the reversion of stoma after assessment by the surgeon. Demographic, clinical characteristics of patients were obtained from the prospectively maintained colorectal cancer database of the Sixth Affiliated Hospital, Sun Yat-sen University. This study was approved by the Medical Ethics Committee of the Sixth Affiliated Hospital, Sun Yat-sen University.

### Stoma status and low anterior resection syndrome

In late 2020, patients were contacted by telephone to complete the questionnaires during a regular visit to the clinic or via mail. If response questionnaires contained missing item(s), the questionnaires were resent and patients recontacted. Patients with no response were contacted every 3 weeks by telephone, and those who made no response by 12 weeks were defined as non-responders.

A persistent stoma was defined by the presence of a stoma 5 years after the index proctectomy or a secondary stoma before death. Patients who underwent upfront APR were excluded. Causes of persistent stoma were determined and classified into three categories: primary diverting stoma not reversed, secondary stoma after recurrence, and secondary stoma without recurrence. The LARS score is a validated instrument for evaluation of bowel dysfunction after sphincter-saving proctectomy, consisting of five items: incontinence of flatus, incontinence of liquid stool, frequency of bowel movements, clustering of stools, and urgency. A total score of 0 to 42 points was classified into no LARS (0–20), minor LARS (21–29), and major LARS (30–42)^13^.

### Outcome of interest

The primary outcome was defined as the combined prevalence of persistent stoma and major LARS.

### Statistical analysis

Patients treated with fluorouracil plus radiation or mFOLFOX6 plus radiation were combined into one nCRT group, compared with the nCT group receiving mFOLFOX6 alone. As the primary hypothesis was that neoadjuvant radiation might be associated with higher prevalence of persistent stoma and major LARS, a logistic regression model was used to test the association of neoadjuvant radiation with the primary outcome. To control for confounding, a threshold of univariate *P* < 0.05 was used to select covariates, which initially included age at proctectomy (continuous), sex, BMI (continuous), clinical tumour, node, and metastasis (cTNM) staging at diagnosis (II/III), tumour height (continuous), height of anastomosis (continuous), diverting ileostomy, anastomotic leak, and time since proctectomy (continuous).

All other evaluations were secondary exploratory analyses. To compare continuous data between treatment groups (nCRT *versus* nCT), the Mann–Whitney *U* test was performed. To compare categorical data, the chi-squared test or Fisher’s exact test was performed. A simple Bonferroni correction for multiple comparisons was used. SPSS^®^ version 25.0 (IBM, Armonk, NY, USA) was used for all statistical analyses. All *P* values were two-sided and *P* < 0.05 was considered statistically significant unless stated otherwise.

## Results

### Patient characteristics

**Fig. 1:**
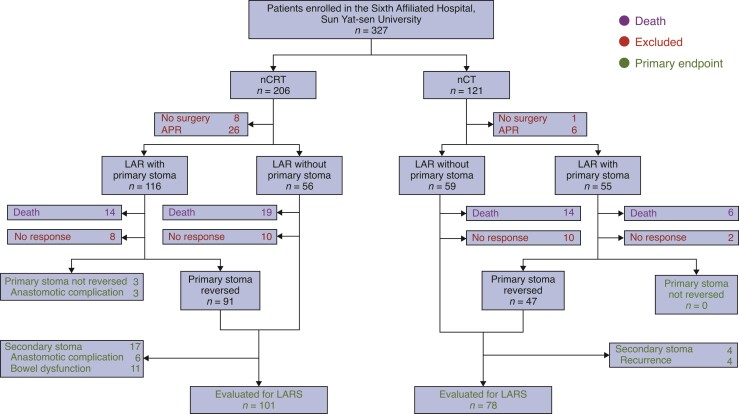
CONSORT diagram of the study selection process^16^ A total of 203 patients were analysed for LARS and stoma status, after exclusion of patients who received APR or no proctectomy, refused to participate, gave no response, or died. APR, abdominoperineal resection; LAR, low anterior resection; nCRT, neoadjuvant chemoradiotherapy; nCT, neoadjuvant chemotherapy; LARS, low anterior resection syndrome.

A total of 327 patients were enrolled at the Sixth Affiliated Hospital, Sun Yat-sen University in the original trial. Follow-up was updated in late 2020, with 274 patients alive at a median follow-up of 83.4 (range, 37.4–121.8) months. A total of 71 patients were excluded due to no proctectomy performed (nine patients), APR (32 patients), refusal to participation or no response (30 patients), with an exclusion rate of 52 of 173 in the nCRT group and 19 of 101 in the nCT group (*Fig. 1*). Clinical characteristics were comparable between the nCRT and nCT groups (*Table 1*), except for primary diverting stoma (77.7 per cent *versus* 57.3 per cent, *P* = 0.002) and anastomotic leak (22.3 per cent *versus* 6.1 per cent, *P* = 0.002). Moreover, responders had statistically significantly higher rate of primary diverting stoma (69.5 per cent *versus* 33.3 per cent, *P* = 0.002) and higher tumours (median 6.3 *versus* 4.8 cm, *P* = 0.005) than non-responders (*Table S1*).

**Table 1.** Clinicopathological characteristics of the 274 patients alive at the second follow-up according to treatment groups

Characteristics	nCRT enrolled (*n* = 121)	nCRT excluded (*n* = 52)	nCT enrolled (*n* = 82)	nCT excluded (*n* = 19)	P*
**Follow-up (months), median (range)**	83 (65–122)	NA	85 (37–120)	NA	
**Age at surgery (years), median (range)**	54 (27–75)	52 (24–77)	55 (23–77)	59 (41–72)	0.846
**Sex**					0.612
Female	41 (33.9)	16 (30.8)	25 (30.5)	6 (31.6)	
Male	80 (66.1)	36 (69.2)	57 (69.5)	13 (68.4)	
**BMI, median (range)**	23 (14–33)	22 (13–31)	23 (17–34)	23 (15–27)	0.890
**cTNM**					0.188
II	23 (19.0)	19 (36.5)	22 (26.8)	5 (26.3)	
III	98 (81.0)	33 (63.5)	60 (73.2)	14 (73.7)	
**Tumour height (cm)†, median (range)**	6.1 (1.7–12.0)	3.6 (1.5–9.0)	6.5 (2.5–12.0)	5.1 (2.0–12.0)	0.130
**Height of anastomosis (cm)†, median (range)**	4.0 (0.5–8.0)	3.0 (1.5–7.5)	4.0 (1.5–12.0)	3.5 (0.5–9.0)	0.112
**Days between radiation and proctectomy, median (range)**	50 (18–163)	50 (39–150)	NA	NA	
**Primary diverting stoma**	94 (77.7)	8 (15.4)	47 (57.3)	2 (10.5)	0.002
**Months before stoma reduction, median (range)**	5 (2–17)	5 (2–11)	5 (2–23)	5 (4–5)	0.389
**Anastomotic leak‡**	27 (22.3)	4 (7.7)	5 (6.1)	1 (5.3)	0.002
Grade A	14 (51.9)	1 (25)	4 (80)	1 (100)	
Grade B	7 (25.9)	3 (75)	1 (20)	0	
Grade C	6 (22.2)	0	0	0	

Values are *n* (%) unless otherwise indicated. *P values were calculated between nCRT group and nCT group. †The distance from anal verge to anastomosis or inferior tumour border. ‡Severity of the anastomotic leak was graded according to the International Study Group of Rectal Cancer classification. nCRT, neoadjuvant chemoradiotherapy; nCT, neoadjuvant chemotherapy; cTNM, clinical staging of tumour, nodes, and metastasis; NA, not applicable.

### Persistent stoma and major LARS

Of the 203 included patients, 24 patients (11.8 per cent) had a persistent stoma, and 48 patients (23.6 per cent) reported major LARS (*Table 2*). Persistent stoma was present in 16.5 per cent patients of the nCRT group, consisting of diverting stoma not reversed (2.5 per cent), and new stoma without recurrence (14.0 per cent). In comparison, persistent stoma was found in 4.9 per cent of the nCT group (*P* = 0.014), all resulting from new stoma after recurrence. The mean time from surgery to secondary stoma was 25.2 months and was comparable between the two groups (*P* = 0.923). For patients without stoma, the nCRT group reported higher prevalence of major LARS than the nCT group (34.7 per cent *versus* 16.7 per cent; *P* = 0.003). The combined prevalence of persistent stoma and major LARS was significantly higher in the nCRT group than in the nCT group (45.5 per cent *versus* 20.7 per cent, *P* < 0.001).

**Table 2.** Comparison of the stoma and low anterior resection syndrome score between the treatment groups

Item	nCRT (*n* = 121)	nCT (*n* = 82)	*P*
Persistent stoma + major LARS	55 (45.5)	17 (20.7)	<0.001
**Persistent stoma**	20 (16.5)	4 (4.9)	0.014
Primary stoma not reduced	3 (2.5)	0 (0)	
Secondary stoma with recurrence	0 (0)	4 (4.9)	
Secondary stoma without recurrence	17 (14.0)	0 (0)	
**Months to secondary stoma, mean(s.d.)**	25.4 (20.2)	24.3 (26.9)	0.923
**LARS score in patients without stoma, mean(s.d.)**	22.3 (12.6)	13.4 (12.6)	<0.001
**LARS severity in patients without stoma**			0.003
Major LARS (30–42)	35 (34.7)	13 (16.7)	
Minor LARS (21–29)	18 (17.8)	8 (10.3)	
No LARS (0–20)	48 (47.5)	57 (73.1)	
Incontinence for flatus			0.147
Never (0)	56 (55.4)	53 (67.9)	
Less than once weekly (4)	27 (26.7)	18 (23.1)	
At least once weekly (7)	18 (17.8)	7 (9.0)	
Incontinence for liquid stool			<0.001
Never (0)	36 (35.6)	51 (65.4)	
Less than once weekly (3)	35 (34.7)	18 (23.1)	
At least once weekly (3)	30 (29.7)	9 (11.5)	
Bowel movement per day			<0.001
>7 (4)	13 (12.9)	4 (5.1)	
4–7 (2)	43 (42.6)	11 (14.1)	
1–3 (0)	35 (34.7)	55 (70.5)	
1 (5)	10 (9.9)	8 (10.3)	
Clustering of stools			<0.001
Never (0)	13 (12.9)	33 (42.3)	
Less than once weekly (9)	19 (18.8)	18 (23.1)	
At least once weekly (11)	69 (68.3)	27 (34.6)	
Urgency			0.012
Never (0)	50 (49.5)	54 (69.2)	
Less than once weekly (11)	23 (22.8)	15 (19.2)	
At least once weekly (16)	28 (27.7)	9 (11.5)	

Values are *n* (%) unless otherwise indicated. LARS score is restricted to patients without persistent stoma. Points are listed with associated responses for each question. LARS, low anterior resection syndrome; nCRT, neoadjuvant chemoradiotherapy; nCT, neoadjuvant chemotherapy; APR, abdominoperineal resection.

### Risk factors for persistent stoma and major LARS

Univariate association with the primary outcome was tested for each clinicopathological factor, with a prespecified threshold of *P* < 0.05 to screen for covariate. Neoadjuvant radiation (*P* < 0.001), tumour height (*P* < 0.001), height of anastomosis (*P* = 0.005), and anastomotic leak (*P* < 0.001) were found to be statistically significant (*Table 3*). Among them, the Spearman correlation coefficient of height of anastomosis with tumour height was 0.738 (*P* < 0.001), while the Spearman correlation coefficient of height of anastomosis with anastomotic leak was 0.007 (*P* = 0.902). To avoid collinearity, tumour height was excluded from the multivariate model, which included neoadjuvant radiation, height of anastomosis, and anastomotic leak, along with age and sex. Multivariate logistic regression demonstrated neoadjuvant radiation (OR 2.20, 95 per cent c.i. 1.10 to 4.40, *P* = 0.027) as an independent risk factor for persistent stoma and major LARS, along with height of anastomosis (OR 0.74, 95 per cent c.i. 0.61 to 0.91, *P* = 0.004) and anastomotic leak (OR 4.97, 95 per cent c.i. 2.24 to 11.05, *P* < 0.001). Of note, in a post hoc analysis, diverting stoma was positively associated with major LARS (33.6 per cent *versus* 8.3 per cent, *P* = 0.001) and was inversely associated with persistent stoma (7.1 per cent *versus* 22.6 per cent, *P* = 0.002).

**Table 3.** Association of factors with persistent stoma and major low anterior resection syndrome of 203 patients in the follow-up

Characteristics	No/minor LARS	Major LARS/persistent stoma	Univariate analysis		Multivariate analysis	
			OR (95% c.i.)	*P*	OR (95% c.i.)	*P*
**Age (years), median (range)**	53 (23–77)	56 (28–75)	1.00 (0.98–1.03)	0.971 0.169	1.01 (0.98–1.04)	0.554
**Sex ratio (M:F)**	84 (61.3):47 (71.2)	53 (38.7):19 (28.8)	1.56 (0.83–2.94):Reference		1.29 (0.64–2.60):Reference	0.486
**BMI, median (range)**	22.6 (14.2–34.0)	22.5 (17.2–33.2)	1.01 (0.91–1.11)	0.898		
**cTNM**				0.472		
II	27 (60.0)	18 (40.0)	Reference			
III	104 (65.8)	54 (34.2)	0.78 (0.39–1.54)			
**Neoadjuvant therapy**				<0.001		0.027
nCT	65 (79.3)	17 (20.7)	Reference		Reference	
nCRT	66 (54.5)	55 (45.5)	3.19 (1.68–6.06)		2.20 (1.10–4.40)	
Tumour height (cm)*				<0.001		
≤3	8 (38.1)	13 (61.9)	Reference			
3–6	41 (56.9)	31 (43.1)	2.11 (1.45–3.06)			
6–9	56 (69.1)	25 (30.9)				
>9	26 (89.7)	3 (10.3)				
Height of anastomosis (cm)*, median (range)	4.0 (0.5–12.0)	3.0 (1.0–7.0)	0.78 (0.66–0.93)	0.005	0.74 (0.61–0.91)	0.004
**Diverting stoma**				0.205		
No	44 (71.0)	18 (29.0)	Reference			
Yes	87 (61.7)	54 (38.3)	1.52 (0.80–2.89)			
**Anastomotic leak†**				<0.001		<0.001
No	123 (71.9)	48 (28.1)	Reference		Reference	
Grade A	8 (44.4)	10 (55.6)	5.07 (2.41–10.67)		4.97 (2.24–11.05)	
Grade B	0	8 (100)				
Grade C	0	6 (100)				
**Follow–up time (months), median (range)**	83 (65–122)	85 (37–120)	1.01 (0.99–1.04)	0.258		

Values are *n* (%) unless otherwise indicated. *The distance from anal verge to anastomosis or inferior tumour border. †Severity of the anastomotic leak was graded according to the International Study Group of Rectal Cancer classification. LARS, low anterior resection syndrome; cTNM, clinical staging of tumour, nodes, and metastasis; nCT, neoadjuvant chemotherapy; nCRT, neoadjuvant chemoradiotherapy.

## Discussion

This study was conducted to test the hypothesis that long-course neoadjuvant radiation is associated with persistent stoma and major LARS in long-term follow-up. This was demonstrated by the higher prevalence of persistent stoma (16.5 per cent *versus* 4.9 per cent) and major LARS (28.9 per cent *versus* 15.9 per cent) in the nCRT group. Multivariate analyses confirmed neoadjuvant radiation, along with height of anastomosis and anastomotic leak, as an independent risk factor for persistent stoma and major LARS.

Many patients with severe and refractory symptoms of LARS eventually resort to permanent faecal diversion^17^, which paradoxically precludes them from further LARS assessment. Thus, the inclusion of these patients in the study was deemed as relevant and necessary. At a median follow-up of 7 years after sphincter-saving proctectomy, 11.8 per cent of patients suffered from persistent stoma, which is comparable to the previously reported 16.7 per cent^18^. A persistent stoma may result from non-closure of primary stoma, or construction of secondary stoma due to postoperative morbidity or recurrence. Although upfront APR also results in permanent stoma, this decision usually depends on patient characteristics and surgeon’s discretion and could not be randomized. For instance, while the Swedish and Dutch trials both reported similar rate of APR between arms with or without preoperative radiation^1,19^, this rate is higher in the nCRT group had higher rate of APR than the nCT group in this cohort (18 per cent *versus* 7 per cent), probably due to the concern of radiation-associated morbidity and bowel dysfunction^9,10,13^. Therefore, patients with upfront APR were excluded to avoid potential confounding factors that might directly affect the primary outcome.

Among patients undergoing sphincter-saving proctectomy, non-closure of primary diverting stoma or construction of secondary stoma was reported in 20 patients in the nCRT group (16.5 per cent) due to anastomotic complication (nine patients) or intractable bowel dysfunction (11 patients), compared with four patients in the nCT group (4.9 per cent) due to local recurrence. A meta-analysis of 8568 patients reported this rate as 19 per cent (range 9.5–27.5 per cent), with variable rates of radiotherapy and follow-up intervals^20^. Neoadjuvant radiation has been demonstrated to reduce local recurrence, though this is not translated into any survival benefit^1^.

These current results of 11.8 per cent persistent stoma and 23.6 per cent major LARS can be compared with a previous follow-up (median, 40 months) of the same FOWARC cohort, which reported 14 (6 per cent) persistent stoma and 119 (51 per cent) major LARS out of 234 patients.^12^ Although some authors suggest that LARS score stabilizes 1 or 2 years after surgery^21^, evidence on serial follow-ups has been limited. Indeed, in a study that followed 78 patients twice a year after surgery, it was found a continuing improvement of LARS for 2 years^22^. Based on the QoLiRECT study, others reported major LARS in 63 per cent at 1 year after surgery and 56 per cent at 2 years^21^. A continued improvement of LARS was also shown in the previous report from our group of 107 patients, with 45 per cent and 24 per cent major LARS at median follow-up of 20 and 38 months respectively^23^. In a longer time span, and in a larger cohort of 282 patients, major LARS were reported in 53 per cent at a median of 6.1 years, and in 49 per cent after 11.1 years^24^. Taken together, it seems that the majority of survivors with major LARS continue to experience symptom relief over the years, whereas an unfortunate minority will have to live with a permanent stoma.

This study reconfirmed neoadjuvant radiation as an independent risk factor for persistent stoma and major LARS, which is corroborated by previous studies^9,10,12^. With no actual benefit for survival and established impact on bowel function^1^, radiation as a standard preoperative treatment requires re-evaluation. For instance, in T2–3 tumours with uninvolved mesorectal fascia, upfront surgery without radiation has been proved to bring satisfactory oncological control^25^. Even in more advanced tumours, neoadjuvant triplet chemotherapy with selective radiation based on radiological response does not seem to compromise oncological outcome^26^.

Of note, diverting stoma was positively associated with major LARS (33.6 per cent *versus* 8.3 per cent), as is observed by other studies^9,12^; however, it was also inversely associated with persistent stoma (7.1 per cent *versus* 22.6 per cent), probably by attenuating the dismal outcome of anastomotic leak. The association of diverting stoma with major LARS and persisting stoma as a combined outcome was therefore statistically not significant. In our opinion, this paradoxical finding supports the relevance of the composite outcome: by excluding patients receiving permanent stoma because of major LARS, long-term follow-up will be biased toward underestimation of functional impairment.

One limitation of this study is the subset analyses of the whole FOWARC randomized clinical trial. However, the analysed subset was drawn from the largest contributing institute of the trial and had similar clinical characteristics with the whole study^14^. Second, death or no response was observed in 25 per cent patients at a median follow-up of 7 years, leading to potential bias. Nevertheless, these patients were similarly distributed between treatment groups and showed similar clinical characteristics with the cohort except for cTNM staging. The response rate was 87 per cent when death cases were excluded, which seemed acceptable for a long-term follow-up. Third, our institute does not routinely perform lateral lymph node dissection, which is recommended by the Japanese guidelines, compared with neoadjuvant chemoradiation by Western guidelines, to reduce local recurrence.

Finally, more than one-third of patients reported major LARS or persistent stoma at a median of 7 years after sphincter-saving proctectomy. Long-course neoadjuvant radiation was independently associated with more major LARS and persistent stoma.

## Supplementary Material

zrac127_Supplementary_Data

## Data Availability

Data are available upon reasonable request.
