# Supplementary material for: Impact of long-course neoadjuvant radiation on postoperative low anterior resection syndrome and stoma status in rectal cancer: long-term functional follow-up of a randomized clinical trial
Source: BJS Open. 2022 Nov 2;6(6):zrac127. doi: 10.1093/bjsopen/zrac127 (PMC10161242; doi:10.1093/bjsopen/zrac127)
Supplement: zrac127_Supplementary_Data [file zrac127_supplementary_data.docx]

Table S1. Clinicopathological characteristics of responders and non-responders.

| Characteristics | Responders  (n=203) | Non-responders (n=30) | *p* |
| --- | --- | --- | --- |
| Age at surgery (years) | 54 (23–77) | 51 (24–77) | 0.390 |
| Gender |  |  | 0.417 |
| Female | 66 (32.5%) | 12(40.0%) |  |
| Male | 137 (67.5%) | 18 (60.0%) |  |
| BMI | 23 (14–34) | 22 (13–31) | 0.075 |
| cTNM |  |  | 0.583 |
| II | 45 (22.2%) | 8 (26.7%) |  |
| III | 158 (77.8%) | 22 (73.3%) |  |
| Tumor height (cm)^*^ | 6.3 (1.7–12.0) | 4.8 (1.5–9.5) | 0.005 |
| Height of anastomosis (cm)^*^ | 4.0 (0.5–12.0) | 3.6 (1.5–9.0) | 0.488 |
| Days between radiation and proctectomy | 50 (18–163) | 50 (25–150) | 0.578 |
| Primary diverting stoma | 141 (69.5%) | 10 (33.3%) | 0.002 |
| Months before stoma reduction | 5 (2–23) | 5 (2–11) | 0.736 |
| Anastomotic leak^†^ | 32 (15.8%) | 5 (16.7%) | 0.899 |

Data are presented as median (range) or n (%). nCRT, neoadjuvant chemoradiotherapy. nCT, neoadjuvant chemotherapy. BMI, body mass index. cTNM, clinical staging of tumor, nodes, and metastasis. N/A, not applicable.

* The distance from anal verge to anastomosis or inferior tumor border.
